# Tracking cells in Life Cell Imaging videos using topological alignments

**DOI:** 10.1186/1748-7188-4-10

**Published:** 2009-07-16

**Authors:** Axel Mosig, Stefan Jäger, Chaofeng Wang, Sumit Nath, Ilker Ersoy, Kannap-pan Palaniappan, Su-Shing Chen

**Affiliations:** 1 Department of Combinatorics and Geometry, CAS-MPG Partner Institute for Computational Biology, 200031 Shanghai, PR China; 2 Max Planck Institute for Mathematics in the Sciences, 04103 Leipzig, Germany; 3 Department of Computer Science, University of Missouri-Columbia, Columbia MO 65211, USA

## Abstract

**Background:**

With the increasing availability of live cell imaging technology, tracking cells and other moving objects in live cell videos has become a major challenge for bioimage informatics. An inherent problem for most cell tracking algorithms is over- or under-segmentation of cells – many algorithms tend to recognize one cell as several cells or vice versa.

**Results:**

We propose to approach this problem through so-called *topological alignments*, which we apply to address the problem of linking segmentations of two consecutive frames in the video sequence. Starting from the output of a conventional segmentation procedure, we align pairs of consecutive frames through assigning sets of segments in one frame to sets of segments in the next frame. We achieve this through finding maximum weighted solutions to a generalized "bipartite matching" between two hierarchies of segments, where we derive weights from relative overlap scores of convex hulls of sets of segments. For solving the matching task, we rely on an integer linear program.

**Conclusion:**

Practical experiments demonstrate that the matching task can be solved efficiently in practice, and that our method is both effective and useful for tracking cells in data sets derived from a so-called *Large Scale Digital Cell Analysis System *(LSDCAS).

**Availability:**

The source code of the implementation is available for download from http://www.picb.ac.cn/patterns/Software/topaln.

## Background

Studying cell motility has become an important factor in understanding numerous biological processes, driven by the rapid development of bio-imaging technology. Accordingly, the computational analysis of live cell video data has attracted significant research activity, with cell tracking as one of the major applications for studying cell motility. Cell motility is crucial for the understanding of phenomena such as tissue repair, metastatic potential, chemotaxis, or the analysis of drug performance [1]; cell migration is also of inherent importance to the immune system, where cell migration towards sites of inflammation engages infectious agents, as well as in embryonic development where migration to distant locations is associated with cell differentiation [2]. Cell tracking has therefore become a major application for biological image processing. As surveyed by [3], this led to a plethora of approaches developed over the past years. While cell tracking algorithms can build on a rich pool of image processing methods that have been developed in the context of other motion tracking problems, biological images contain their own intricacies. Often, bioimage data are captured in order to quantify phenomena such as cell division or cell fusion. However, such events are difficult to recognize computationally, in particular when dealing with 2D images of a tissue or cell culture that hides essential 3D information and contains a large number of cells. In fact, in the presence of cell division, the number of objects to be tracked can eventually double within the course of one captured video sequence. Further challenges in biological image processing are inherently low contrast images and cells changing their shape or momentum abruptly.

Given the current state of the art in image processing, cell tracking under noise-free and high-contrast circumstances, such as fluorescently labelled bacteria, is a tractable task. However, in most cases, we will see one or more of the above challenges complicating the problem. For these video sequences cell tracking remains a formidable problem. To address this problem, we follow a commonly used two-stage approach: In the first stage, we apply a segmentation procedure on each individual frame, where we rely on a previously established image processing procedure. In the second stage – the so-called *linking *stage – our newly developed topological alignment links segments between each frame *i *and the next frame *i *+ 1. In order to trace one cell, we match a *set of segments *in frame *i *onto another set of segments in frame *i *+ 1. Our matching approach indeed allows to do this for several cells simultaneously, i.e., matching *several *sets of segments onto other sets of segments in the next frame. The many-to-many matching underlying our approach to the linking problem is much more flexible than existing approaches, which essentially rely on one-to-one matchings.

We achieve the generalization to many-to-many matchings through arranging the segments in a hierarchy using single linkage clustering; then, we find an optimal "bipartite matching" between the two hierarchies, which can indeed be viewed as a generalization of bipartite matchings in the classical sense. We approach this problem using a linear programming formulation. Being based on overlap of segment groups in the two frames, our approach can be seen as a "topological alignment" between two images. The idea behind our approach is that our novel topological alignment procedure allows to identify cell division and fusion events, and in particular can distinguish them from from errors produced by the segmentation procedure; for dealing with low contrast images and shape-changing cells, on the other hand, we rely on the flux tensor method from Palaniappan et al., which has been shown to be sufficiently robust against such effects in [4].

### Related Work

Existing approaches to cell tracking, as surveyed in [3] and [5], essentially come in two flavors, namely segmentation based methods and segmentation-free approaches. Following the terminology in [6], segmentation-based approaches – including the one presented in this paper – work in two stages: first, a *detection *step is conducted, which aims to identify individual cells in every single frame. This is typically achieved through a segmentation procedure, involving techniques such as thresholding or level-set-methods [1,7-9]. Recently, Palaniappan et al. [4,10] obtained more robust segmentations by combining level-set methods with the so-called flux-tensor. The second stage then performs the *linking *of consecutive frames by assigning the cells identified in frame *i *to the cells identified in frame *i *+ 1. For instance, the authors in [11,12] determine the assignment that best matches the distances traveled by each individual segment. A possible refinement of this approach is the inclusion of probability distributions for the anticipated positional changes [13,14]. Other authors employed graph-theoretical methods for resolving ties in case of multiple candidates that could equally likely be linked to the same object [15]. Compared to the approach proposed by us, all these approaches rely on mapping segments one-to-one between consecutive frames, making it difficult to handle events such as cell division, cell fusion, or over-segmentation. Our topological alignment approach addresses the linking problem by allowing many-to-many mappings between segment sets in different frames.

Notwithstanding the advantages achievable by advanced methods for solving the linking problem, segmentation-free approaches as an alternative contributed major progress in the field recently. Among those, deformable models – closed curves in 2-D, or surfaces in 3-D that evolve iteratively around the boundaries of objects [3] – have taken center stage in cell segmentation and tracking. Due to the flexibility of combining image characteristics with prior knowledge, deformable models have become very popular in medical imaging [16]. [3] distinguish between two main categories of deformable models, namely explicit functions (e.g., [17]) and implicit models (e.g., [18]). Among deformable models, *active contours *have become very popular [19-23] and demonstrated particularly successful recently.

## Methods

Our topological alignment approach addresses the linking problem and hence builds upon a segmentation procedure that is applied to each frame individually. We segment the images using the approach from [4], which combines flux tensors for detection of moving objects with a multi-feature level-set method. This approach allows extraction of more compact boundaries and improved localization of moving non-homogeneous objects. While providing good results on video sequences with reasonably high contrast and low noise levels, the performance of flux-tensor level-set segmentation weakens as contrast decreases and noise increases – a phenomenon that naturally occurs for any segmentation procedure as contrast gets too low or noise too high. In fact, we often observe the phenomenon of *over-segmentation*, i.e., a single cell is represented by several segments; less frequently, one can also observe under-segmentation, i.e., several cells identified as one segment. As the number and density of cells in a cell culture increases, it can be expected that any segmentation procedure will be more and more likely to produce such over- or under-segmentations.

**Figure 1:**
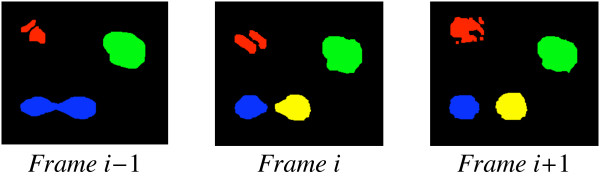
**Artificially produced segmentations representing a pseudo-division *(red) *and a cell-division *(blue, yellow) *over a sequence of three frames: The segments marked in red split into several parts by the segmentation procedure, hence constituting a *pseudo-division***. The blue segment, on the other hand, actually splits into two cells.

As over- or under-segmentation appear to be essentially unavoidable side-effects of segmentation, the idea of our topological alignment procedure is to compensate these by aligning the segmentations of each two consecutive frames in the video sequence; the alignment aims to map *sets of segments *in the first frame onto sets of segments in the second frame, maximizing the overlap between the two frames. The main challenge therein is to distinguish biological cell division from *pseudo-division*, i.e., erroneous splits of one cell into several segments, as depicted in figure 1. Pseudo-division is common due to phenomena such as noise in the underlying images. Distinguishing cell division events from pseudo-division, in fact, is the major challenge addressed by our alignment procedure.

The commonly observed phenomenon of pseudo-division leads us to formalize the problem of aligning two consecutive frames as a generalized assignment problem. Formally, we capture this as a partitioning problem: We identify the segmentation of the first image into *m *segments with an index set *P *= {1,..., *m*}, and the segmentation of the second image into *n *segments with an index set *Q *= {1,..., *n*}. Now, alignments between these sets can formally be introduced through partitioning *P *and *Q *into an equal number of subsets: ℓ denoting an integer and *M *a (finite) set, we say that a family *m*_1_,..., *m*_ℓ _of subsets of *M *is an ℓ-*partitioning of M *iff *M *= *m*_1_∪ ⋯ ∪ *m*_ℓ _and *m*_*i *_∩ *m*_*j *_is empty for any *I *≠ *j*. Given an integer ℓ along with the segment indices *P *and *Q*, we are now interested in "simultaneously" partitioning *P *and *Q *into ℓ segments each, so that *P *= *p*_1_∪ ⋯ ∪ *p*_ℓ _and *Q *= *q*_1_∪ ⋯ ∪ *q*_ℓ_; for each *i*, the segments in *p*_*i *_are identified with the segments in *q*_*i *_as one cell. The generalized assignment problem now is to find a maximum weighted ℓ-partitioning (with respect to a suitable weighting scheme); we will treat ℓ as a variable that is to be maximized along with the actual partitioning.

### Linear Programming Formulation

A central point in our assignment procedure is to assign a weight *w*(*p, q*) to matching segment sets *p *⊆ *P *onto *q *⊆ *Q*. Here, segment sets *p *and *q *that are likely to represent the same cells in both frames should receive a high score and vice versa. We measure weights based on the "relative overlap" of the convex hulls of *p *and *q*. Correspondingly, we identify *p *⊆ *P *with the convex hull of the area covered by all segments in *P*, i.e. , where *α *(*x*) denotes the area covered by segment *x *and  denotes the convex hull of a set *X *of points in the plain. Assuming that cells move moderately between two consecutive frames, we assign the relative overlap of *p *and *q *as their weight, formally defined as(1)

Naturally, sets of segments that achieve a relative overlap close to 1 should more likely be considered as one cell, while overlap close to 0 indicates segment sets that do not constitute one cell.

Based on these weights, we can now further formalize our notion of a topological alignment. We denote **P**_ℓ_(*M*) for the set of all ℓ-partitionings of a finite set *M*; note that given a partition *S *∈ **P**_ℓ_(*M*), we consider *S *as a family of sets and hence can identify the ℓ subsets by writing *S *= (*S*_1_,..., *S*_ℓ_). This allows us to state our alignment as finding those partitionings *S *and *T *that realize the maximum in the target function(2)

Optimizing over the undoubtedly huge space of all ℓ-partitionings of *P *and *Q *requires more attention to be tractable in practice. Our approach is to first develop an integer linear programming (ILP) formulation. While in general, this formulation involves a doubly exponential number of variables and constraints, we introduce heuristics that will choose a quadratic number of variables to make the problem solvable in practice through state-of-the-art ILP solvers.

The general linear programming formulation indeed is quite straightforward. For each *p *⊆ *P *and *q *⊆ *Q*, we introduce a binary variable *X*_*p*, *q*_, where *X*_*p*, *q *_= 1 if and only if *p *= *S*_*i *_and *q *= *T*_*i *_for some *i *in the optimal partitionings *S *∈ **P**_ℓ_(*P*) and *T *∈ **P**_ℓ_(*P*). This immediately yields the target function for the integer linear program, namely(3)

To maximize over valid partitionings only, we need to avoid subsets *p, p' *of *P *with non-empty intersection being chosen (and, correspondingly, overlapping subsets from *Q*). This can be done by introducing constraints(4)

whenever *p *∩ *p' *≠ ∅ or *q *∩ *q' *≠ ∅. A remarkable property about the constraint matrix resulting from Eq. (4) is that it is *totally unimodular*, so that the linear programming relaxation of the ILP will have an optimal solution that is integral [24]. To see total unimodularity of the constraint matrix *C*, note that *C *is the incidence matrix of the bipartite graph *B *= (*L *∪ *R*, *E*), where *L *= {*pp'*|*p*, *p' *⊆ *P*} and *R *= {*qq' *| *q*, *q' *⊆ *Q*}, and *E *introduces one edge for each constraint, namely

**Figure 2:**
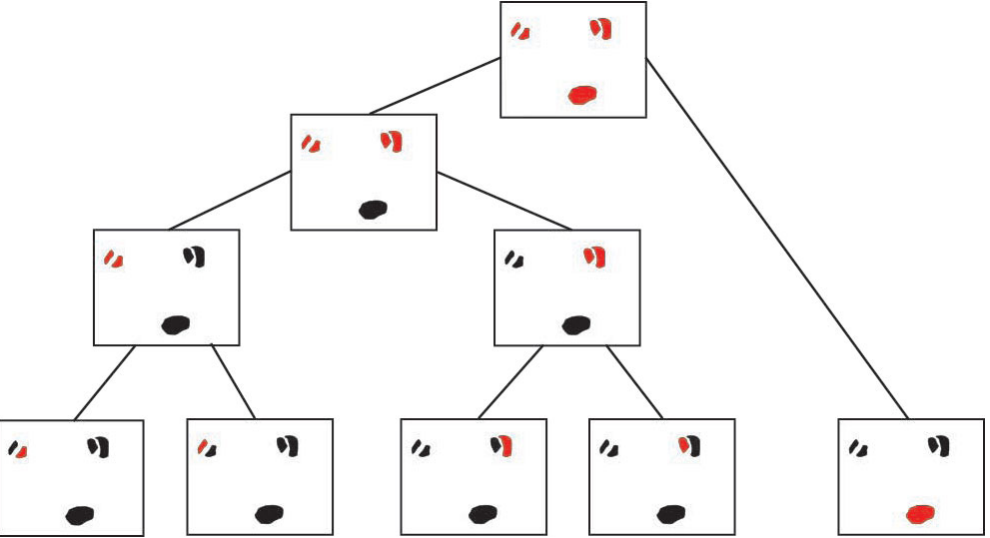
**Reducing the number of variables in the integer linear program from exponential to quadratic through hierarchically clustering the segments: Introducing one variable for each vertex in the hierarchy introduces variables for all those sets of segments that are "close to each other"**.

As being the incidence matrix of a bipartite graph, *C *is in particular totally unimodular [24]. Despite the convenient property of unimodularity, the above linear programming formulation is not practical in general: both the number of variables and the number of constraints are inherently exponential in the number of segments in the two input images. To make it suitable for practical purposes, we deal with a restricted version of the original partitioning problem that leads to a *tree assignment problem*. The key observation for this restriction is that if we identify several segments as one cell, these segments should be "close to each other". Hence, it is reasonable to deduce those sets of segments for which variables should be generated from clustering the segments. In fact, performing single linkage clustering on the segments allows us to introduce one variable for each node of the clustering hierarchy, representing the set of all leaves underneath that node as indicated in figure 2. Since the single linkage tree for *n *segments has 2*n *- 1 nodes, we obtain a quadratic number of variables in our relaxed linear program, which can be solved using the standard simplex algorithm as implemented in state-of-the-art solver software. Note that unimodularity makes the tree assignment problem solvable in polynomial time.

### Tracking cells across whole video sequences

So far, we have only dealt with tracking cells between consecutive frames. To make sure that we can track cells not just across two consecutive frames, but through a complete video sequence, we need to "carry cell identities" through time. To this end, we introduce one color for each set of segments that has been identified as one cell. When aligning frame *i *with frame *i *+ 1, we carry as much color information as possible from the previous alignment of frame *i *- 1 with frame *i*. To do so, we essentially need to deal with two different partitionings: The cells *C*_1_,..., *C*_*k *_in frame *i*, as identified from the alignment with frame *i *- 1, and the cells *D*_1_,..., *D*_ℓ _in frame *i*, as identified from the alignment with frame *i *+ 1. While each of the cells *C*_*μ *_has already received a color in the previous stage, the cells *D*_*ν *_are to be colored. Note that with each *C*_*μ *_and each *D*_*ν*_, we can associate the corresponding set of pixels in the segmentation, which allows us to compute the convex hulls  and  of each cell, along with their relative overlap as defined in Eq. 1. In other words, we can set up a bipartite graph with *k *vertices in one layer and ℓ vertices in the other layer, and relative overlap scores as weights on the edges. On the basis of this graph, we can compute a straightforward maximum-edge-weighted bipartite matching. Whenever vertex *μ *is matched with vertex *ν*, *D*_*ν *_receives the same color as *C*_*μ*_; unmapped vertices *D*_*ν *_correspond to cells either resulting from a cell division or entering the image from the side and receive a new, previously unassigned color.

Across all *n *frames of a cell video, the above construction leads to a multi-partite graph with *n *layers, obtained by "concatenating" the bipartite graphs. This graph corresponds to the *cell connection graph *as introduced in [25]. In [25], each vertex corresponds to one segment; in our approach, however, one vertex in the connection graph represents several segments. In the current implementation, the cell connection graph is the final outcome of the cell tracking procedure. As a future extension, post-processing the connection graph may indeed to further improvements, since it allows to take a more global view at the video sequence for spotting over- or under-segmentation that occur within one individual or a few consecutive frames only.

## Results

### Comparing output with ground truth

Diverse performance measures for cell tracking have been used [26-29], often tailored to measure performance specific for a particular application context. In our setting, we primarily aim to measure the quality of the topological alignments computed in the linking stage. Essentially, measuring the quality of an automated cell tracking procedure requires two components, namely a *ground truth *annotation and a *distance measure *or scoring scheme to compare a computationally produced tracking with the ground truth annotation.

Following the two-step nature of segmentation-based approaches, we deal with two levels ground-truth annotation, the *segmentation annotation *and the *partitioning annotation*. A segmentation annotation provides a polygon around each cell as an approximation of the cell's boundary. A partitioning annotation, on the other hand, annotates the segmentation produced by the segmentation algorithm, in our case the output of the method from [4]. Here, the annotator assigns each segment of the input segmentation to one of the cells labelled in the first step by coloring the segments; segments receive the same color if and only if they belong to the same cell. Since we aim to judge the quality of topological alignments for the linking problem, we assess quality on the basis of a partitioning annotation. On our context, the purpose of the segmentation annotation is mainly to have a comprehensible basis for a reproducible partitioning annotation.

**Figure 3:**
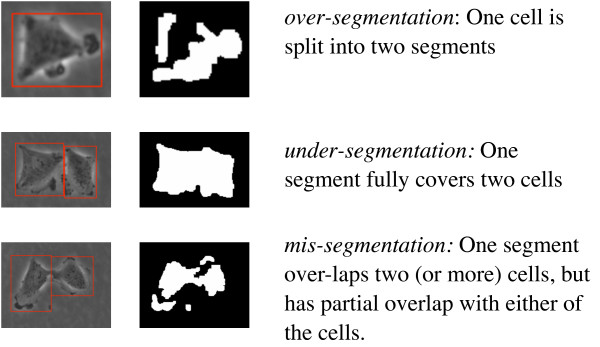
**Three types of errors can occur at the level of the segmentation**. Quantifying these errors for a ground truth data set requires manual annotation.

**Figure 4:**
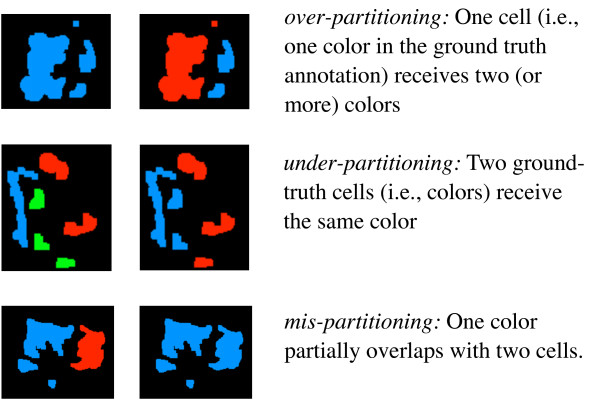
**Three types of errors can occur at the level of the partitioning**. The images on the left indicate the ground truth annotation, while the images on the right represent partitioning obtained computationally. As shown in figure 5, we can recognize these errors from connected components of the *overlap graph*.

Both levels of annotation unveil different types of errors in the corresponding stages of cell tracking, as shown in figures 3 and 4. Segmentation errors have some influence on the partitioning annotation: while over-segmentation is compensated for in the partitioning annotation, both mis-segmentation and under-segmentation lead to a certain loss of information. For under-segmentations, one cell needs to be dropped; for mis-segmentations, we chose to segment the actual cells as far as possible and annotate those segments overlapping more than one cell in a separate color. This way, mis-segmentation resulting from the segmentation procedure will be recognized as under-partitioning on the partitioning level.

**Figure 5:**
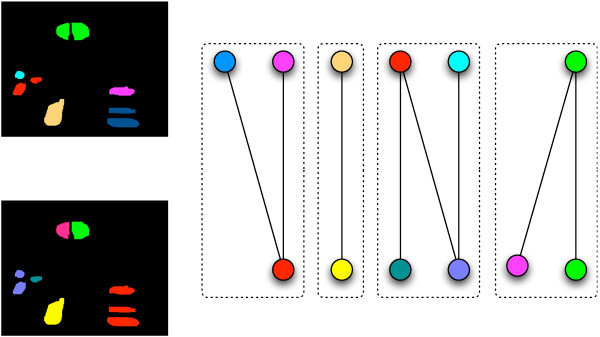
***Left*: A ground-truth partitioning (*top*) and a computationally determined partitioning (*bottom*)**. *Right*: The corresponding overlap graph with four connected components. In the overlap graph, we introduce one vertex for each ground-truth cell (i.e., color), which constitutes the top layer. In the bottom layer, we introduce one vertex for each color in the computed partitioning. We introduce an edge between two vertices if and only if at least one segment receives the corresponding colors in the ground-truth partitioning and the computed partitioning, respectively. Connected components that consist of one edge only constitute correct assignments. Those components involving only one vertex on either side represent over- or under-partitionings, respectively. Connected components with more than one vertex on both sides constitute mis-partitionings.

Note that partitioning errors can be quantified easily by computational means once a ground truth data set is available. To determine the different partitioning error types, we classify the connected components of a certain bipartite graph, the so-called *overlap-graph*, as shown in figure 5.

### Application to LSDCAS data set

We applied our method on a live cell video produced by the *Large-Scale Digital Cell Analysis System *(LSDCAS) [30]. The sequence of 363 images in this video (see http://www.picb.ac.cn/patterns/Supplements/topaln) was segmented using the flux-tensor based approach described in [4]. To obtain a ground truth, we annotated the original images manually using the Viper toolkit [31,32]. Based on this annotation of the raw images, we manually annotated the flux-tensor based segmentation by coloring the segments using a simple drawing program. This finally allowed us to compare the results of the topological alignment with the annotated segmentation by counting over-, under-, and mis-partitionings.

**Figure 6:**
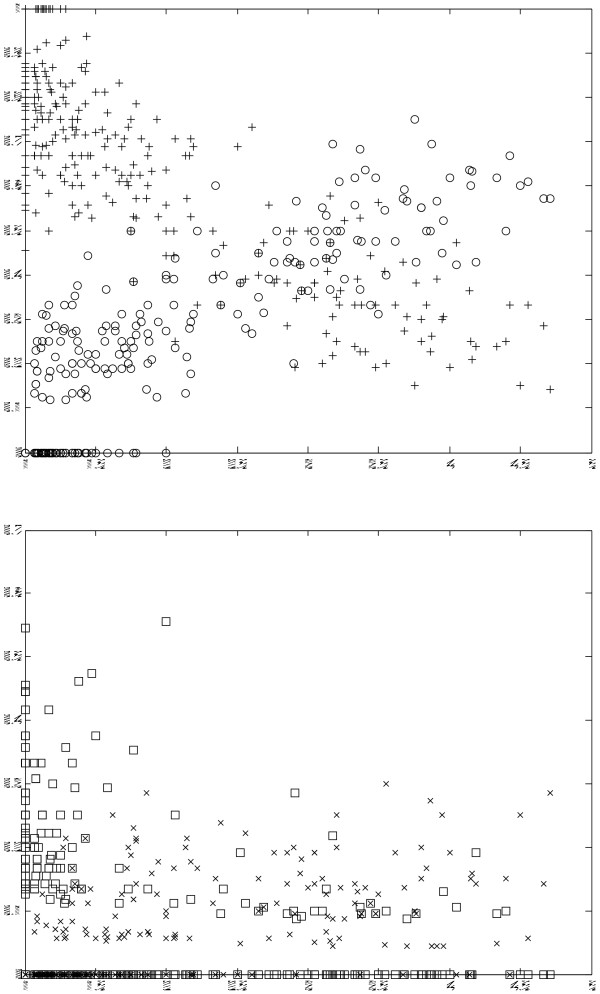
***Top*: percentage of correctly identified cells vs**. LOS (*crosses*) and percentage of mis-segmented cells vs. LOS (*circles*). While the ratio of correctly identified cells decreases proportional to LOS, mis-segmentations increase correspondingly. *Bottom*: ratio of over-segmented cells vs. LOS (*crosses*) and ratio of under-segmented cells vs. LOS (*s*quares), which are much weaker – if at all – correlated with LOS.

Not surprisingly, the flux-tensor segmentation tends to over-segment cells, i.e., split each cell into several segments, while under-segmentations are observed less frequently. As the level of over-segmentation increases, the topological alignment task naturally gets more challenging and prone to producing the error types described above. This motivates us evaluate our cell tracking results in relation to the *level of over-segmentation *(LOS) of each frame. The LOS of a single frame is naturally defined as the number of segments divided by the number of cells in the frame. Note that the LOS of each frame can be computed in a straightforward manner once a ground-truth annotation and a topological alignment are available. As it turns out, the LOS varies significantly across the roughly 400 frames of our reference data set, ranging between 1 and 4.5. In general, the rough proportionality between LOS and quality of topological alignment output observed in figure 6 suggests that input segmentations with a lower LOS will lead to alignments with lower degrees over- or under-partitioning.

### Implementation

**Figure 7:**
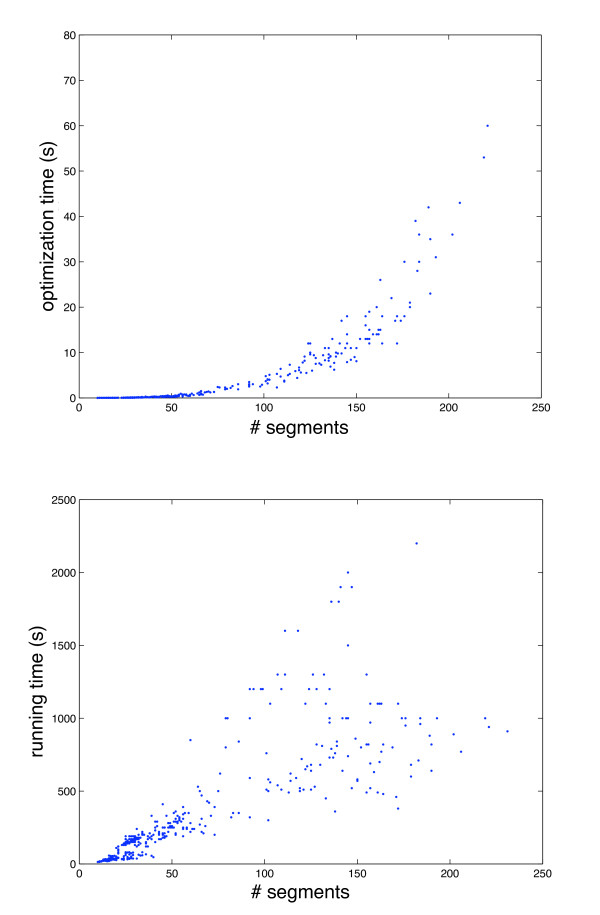
**Running times of aligning two frames, in dependence of the total number of segments in the two frames**. Only a fraction of the overall time is spent for solving the ILP (*top*), while the overall time (*bottom*) is dominated by setting up the linear program, in particular computing the weights.

We implemented the algorithm in C++ using the CPLEX solver to solve both the topological alignment ILP and the bipartite matching for obtaining the cell connection graph. Convex hulls for obtaining the weights are implemented using a standard Graham scan. Single-linkage clustering requires an initial computation of the minimal distances between each pair of segments, requiring a fast algorithm for finding *bichromatic closest pairs*. Here, we rely on a non-optimal algorithm that works sufficiently fast on the given data set rather than recently developed sophisticated approaches [33]. Running times for aligning frame pairs containing between 7 and 99 segments are always observed below one hour on a 2.0 GHz Intel Xeon processor with 32 GByte main memory running CPLEX Version 10.2. We used the default settings of the CPLEX mixed integer programming solver. Changing these default settings did not result in significantly improved running times, which might be attributed to the unimodularity of the constraint matrix. All solutions were reported optimal; small instances with a dozen or less segments are typically solved within seconds or few minutes. As shown in figure 7, the running time is overwhelmingly dominated by computing the convex hulls for the weights of the integer linear program variables rather than solving the ILP itself.

## Discussion

As we have demonstrated, our topological alignment approach improves the performance of segmentation-based cell tracking approaches by explicitly taking into account the inherent problems of over- and under-segmentation, while still allowing the detection of cell division. Naturally, our approach can be used to post-process the output of any segmentation-based cell tracking procedure, and can in principle also be used to improve cell tracking results obtained from a segmentation-free procedure. Our results suggest that indeed significant improvement can be achieved as long as the degree of over-segmentation remains within reasonable bounds.

While the major goal of this contribution is to demonstrate the ability of the topological alignment approach to improve cell tracking quality, several tracking related issues leave space for improvement. A major difficulty is to avoid under-segmentation in the input segmentation, since under-segmented cells cannot be resolved into several cells by our approach. To overcome this, two developments are currently on their way. First of all, we intend to use a hierarchical segmentation rather than a fixed segmentation as an input to the topological alignment. This is a natural choice that relieves us from "artificially" imposing a hierarchy on a fixed segmentation using single-linkage clustering. Also, a number of hierarchical segmentation methods such as level-set-trees have been developed and need only minor adaptation to integrate with our topological alignment procedure. A second promising improvement is to post-process the cell connection graph after performing topological alignments of all consecutive frames. The cell connection graph in principle allows to "look across several frames" and hence distinguish over-partitioning from cell division on a larger time-scale.

In principle, further improved can be obtained by taking into account the size or shape of cells. Such aspects can easily be incorporated in the linear programming formulation, for instance by adjusting weights or eliminating variables. While we intentionally avoided this in the present work in order not to introduce further parameters or even modelling (largely unexplored) shape constraints of the displayed cells, this might be helpful in future applications.

From an algorithmic point of view, the ILP formulation allows to find solutions quickly in practice, even without tuning any parameters or settings of the ILP solver. For future applications, the unimodularity of the integer linear programming formulation suggests to exploit this property more systematically, and eventually obtain an efficient algorithm for the topological alignment problem with better guaranteed bounds on the running time.

## Competing interests

The authors declare that they have no competing interests.

## Authors' contributions

AM conceived and coordinated the research. SJ, WC and AM developed measures for validating results, implemented topological alignments, and drafted the manuscript; SN and IE contributed weighting schemes; KP and SC initiated the application of topological alignments to cell tracking and coordinated the study jointly with AM. All authors read and approved the final manuscript.
